# The Impact of Bilayer Rigidity on the Release from Magnetoliposomes Vesicles Controlled by PEMFs

**DOI:** 10.3390/pharmaceutics13101712

**Published:** 2021-10-16

**Authors:** Jordan Trilli, Laura Caramazza, Patrizia Paolicelli, Maria Antonietta Casadei, Micaela Liberti, Francesca Apollonio, Stefania Petralito

**Affiliations:** 1 Department of Drug Chemistry and Technologies, Sapienza University of Rome, 00185 Rome, Italy; jordan.trilli@uniroma1.it (J.T.); patrizia.paolicelli@uniroma1.it (P.P.); mariaantonietta.casadei@uniroma1.it (M.A.C.); stefania.petralito@uniroma1.it (S.P.); 2 ICEmB at DIET, Sapienza University of Rome, 00184 Rome, Italy or laura.caramazza@iit.it (L.C.); micaela.liberti@uniroma1.it (M.L.); 3 Center for Life Nano- & Neuro-Science, Fondazione Istituto Italiano di Tecnologia (IIT), 00161 Rome, Italy

**Keywords:** magneto mechanical trigger, magnetoliposomes, on-demand drug delivery, magneto nanoparticles, pulsed electromagnetic fields, non-thermal magnetic field

## Abstract

Stimuli-sensitive nanocarriers have recently been developed as a powerful tool in biomedical applications such as drug delivery, detection, and gene transfer techniques. Among the external triggers investigated, low intensity magnetic fields represent a non-invasive way to remotely control the release of compounds from a magneto-sensitive carrier. Magnetoliposomes (MLs), i.e., liposomes entrapping magnetic nanoparticles (MNPs), are studied due to their capacity to transport hydrophobic and hydrophilic agents, their easy production, and due to the ability of MNPs to respond to a magnetic actuation determining the triggered release of the encapsulated compounds. Here we investigated the design and optimization of the MLs to obtain an efficient on-demand release of the transported compounds, due to the magneto-mechanical actuation induced by applying low-intensity pulsed electromagnetic fields (PEMFs). In particular we studied the effect of the bilayer packing on the ability of MLs, with oleic acid-coated MNPs encapsulated in the bilayer, to respond to PEMFs application. Three kinds of MLs are produced with an increasing rigidity of the bilayer, defined as Liquid Disorder, Liquid Order, and Gel MLs and the delivery of a hydrophilic dye (as a model drug) is investigated. Results demonstrate the efficacy of the magnetic trigger on high-ordered bilayers, which are unable to dampen the perturbation produced by MNPs motion.

## 1. Introduction

Since their discovery [1], liposomes have gained great interest due to their biocompatibility, the possibility to adjust their size and membrane composition and the ability to encapsulate compounds with different chemical properties into their internal core or in their lipid bilayer for drug delivery and gene therapy purposes [2]. Liposomes can easily be engineered with a wide variety of functional materials to achieve specific purposes. Chemical modification of the surface can be used to introduce specific ligands for the active targeting of liposomes [3]. On the other hand, they can be modified to confer stimulus-responsive properties, whereby the encapsulated drug release can be achieved when internal (such as pH, redox and specific enzymes) or external stimuli (temperature, light, magnetic field, electric field or ultra-sound) are present [4,5].

Among all investigated stimuli-responsive liposomes, early efforts are focused on the use of a magnetic field as an external trigger and using magnetic nanoparticles (MNPs) to control drug delivery from liposomes [6,7]. The combination of MNPs and liposomes produces the so-called magnetoliposomes (MLs). Usually, magnetic hyperthermia is used to trigger release of drugs from MLs [8]. This approach exploits MNPs as nano-heaters to produce a modification in the bilayer physical state and consequently to the permeability of MLs. When an external heating source is used as a trigger, a convenient strategy is to utilize thermo-sensitive nanocarriers in order to maximize the release. For this reason, the successful application of the magneto-thermal approach strictly requires the use of temperature-sensitive liposomes, with a main phase transition temperature (Tm) in the range of 39 °C to 42 °C [9,10].

More recently, attention has begun shifting towards a different actuation of MLs. Specifically, it has been observed that it is possible to obtain controlled drug release from MLs even in non-heating conditions, using alternating magnetic fields (AMF) of appropriate low intensity and frequency or pulsed electromagnetic fields (PEMFs) [11,12,13,14]. Under these conditions, MNPs act as magneto-mechanical actuators. This approach represents an interesting alternative to the thermo-magnetic actuation: the tissues are affected neither by the temperature nor by the eddy currents induced by the field, thus preventing damage and safety secondary effects [15]. In this case, the physical mechanism behind the magnetic approach does not involve heat dissipation by the MNPs, but rather a mechanical destabilization of lipid membranes of MLs.

In particular, this mechanism has been demonstrated by the authors in the past using hydrophilic MNPs entrapped in the aqueous core of MLs: in order to exclude the role of minimal temperature increase due to insignificant heating, MLs were produced with the bilayer characterized by a gel ordered state with a Tm above 50 °C [13,14]. After the magnetic field application, results demonstrated that the observed increase of dye release was undoubtedly not related to the liposomal membrane transition, from gel to liquid crystalline state, but it was due to the magnetic-impelled motions of the entrapped MNPs. It was shown that the co-entrapment of MNPs and hydrophilic compounds in the internal space of liposomes could reduce the entrapment itself, due to their co-presence within the same volume.

To overcome this issue, we followed an alternative approach, based on the use of MLs containing hydrophobic MNPs within their bilayer. In this case it is crucial to understand whether the MNPs can move in the lipid microenvironment under the effect of a non-thermal magnetic field [16,17]. In this regard it is important to understand the influence of phospholipid composition, lateral packing, and organization on the effectiveness of magneto-mechanical actuation. Indeed, depending on the type of lipids used to produce MLs, the responsiveness of the carrier could be enhanced or reduced. Both responses in principle may be possible: the movement of hydrophobic MNPs embedded in a lipid bilayer in a gel state could disrupt the rigid ordered packing of the lipids, triggering the cargo release, whereas a liquid-crystalline membrane could be able to heal the defects created by mechanical motion of the MNPs, thus resulting in a decreased release of the cargo [18].

The goal of this work is to obtain a release from MLs with hydrophobic embedded MNPs when undergoing non-thermal PEMFs stimulation. To this aim, we characterized bilayers with various degrees of order, investigating the dependence of the magnetic field effect on the structural mechanical properties of the nanocarriers. The study was performed using MLs formed by saturated or unsaturated phosphatidylcholine. The liposomes structural changes were studied using a Dynamic Light Scattering (DLS), and the release was monitored in time using a fluorescent dye as a hydrophilic model drug.

The final aim of the work is, therefore, to understand if magneto-mechanical actuation due to PEMFs can be effective with the MNPs within the MLs bilayer and under which phospholipid composition. Specifically, this work will address the role of gel and liquid phospholipid in PEMFs destabilization of vesicles for the design and optimization of stimuli-sensitive MLs.

## 2. Materials and Methods

### 2.1. Materials

Egg phosphatidylcholine (Egg-PC) Lipoid 80 E, Hydrogenated phosphatidylcholine from soybean (HSPC) and Phospholipon 90 H were kindly offered by AVG srl (Bollate, MI, Italy). Cholesterol (Chol), 4-(2-hydroxyethyl)-1-piperazine ethanesulfonic acid (HEPES), 5-(6) carboxyfluorescein [5-(6) CF], Triton X-100 (TX-100) and Sephadex G-50 medium grade were purchased from Sigma Aldrich (Saint Louis, MO, USA). Chloroform (CHCl_3_) was obtained from Merck (Kenilworth, NJ, USA). Diisopropyl ether was supplied by CARLO ERBA Reagents (Cornaredo, MI, Italy). Fe_3_O_4_ magnetic nanoparticles (MNPs) coated with oleic acid were purchased from Das Nano (Tajonar, NA, Spain). MNPs are ultra-monodispersed, with particle diameter distribution centered at 3.8 nm.

### 2.2. Liposome Preparation and Characterization

Conventional liposomes (i.e., without MNPs, in the following CLs) and MLs were prepared using two different techniques, thin layer evaporation (TLE) and reverse phase evaporation (REV) [19,20], in order to completely optimize the design of stimuli-sensitive nanocarriers. For TLE procedure see Supplementary Materials Figure S1.

For the REV procedure, liposomes were prepared as described by Szoka et al. [20]. Briefly, we dispersed in 3 mL of chloroform/diisopropyl ether a 1:1 *v*/*v* mixture (supplemented with 0.5 mg of MNPs for MLs) respectively: 40.0 mg of HSPC (for Gel Liposomes) or Egg-PC (for Liquid Disorder Liposomes) and we added 4.0 mg of Chol for Liquid Order Liposomes. Successively, the solution was mixed with 1 mL of 20 mM 5-(6) CF solution in HEPES buffer (10 mM, pH = 7.4) and was sonicated, above lipid Tm, until a stable emulsion was obtained. The organic solvents were evaporated on a rotary evaporator until a viscous gel was formed. The volume was adjusted to 3 mL by addition of a 20 mM 5-(6) CF solution and the viscous gel was sonicated, above lipid Tm, up to the formation of a homogeneous dispersion. The unencapsulated fluorescent dye was then removed by a size exclusion chromatography (SEC) with a Sephadex G-50 column. After the preparation, a physico-chemical characterization of the MLs and CLs was carried out in terms of sizing (hydrodynamic diameter and size distribution) with a Zetasizer Pro (Malvern Instruments Ltd., Malvern, United Kingdom) at the constant temperature of 25.0 ± 0.1 °C, in order to ensure homogeneous size distribution.

### 2.3. Release Measurement from Liposomes

The release from CLs and MLs at PEMFs exposure conditions was determined by monitoring 5-(6) CF release, based on the increase of fluorescence intensity due to the de-quenching of the released dye. MLs and CLs after SEC purification were diluted (1:25) and at specific times the fluorescence intensity was monitored.

In vitro dye release from MLs and CLs was performed in single read modality, with a spectrofluorometer (FL 6500 Perkin Elmer, Waltman, MA, USA), using the λ_ex_ = 492 nm and λ_em_ =512 nm, as previously determined. In order to evaluate the total amount of 5-(6) CF entrapped, the liposomal vesicles were completely destroyed by adding a lytic concentrated solution of non-ionic detergent TX-100 (30% *w*/*v*). The released percentage of hydrophilic dye was calculated with the following equation:

(1) $$5\text{-}\left(6\right)\;CF\;release\;\left(\%\right)=\frac{I_{t}-I_{0}}{I_{tmax}-I_{0}}\times 100\;$$

where, *I*_0_ is the initial fluorescence intensity of 5-(6) CF in the bulk suspension, *I_t_* represents the fluorescence intensity recorded at specific times and *I_tmax_* corresponds to the maximum intensity after the liposomes’ lysis with TX-100. All the measurements were performed in triplicate and the results are reported as mean ± standard deviation.

### 2.4. PEMFs Exposure Setup and Exposure Protocol

The PEMFs magnetic source is a commercial device (I-ONE, IGEA, Carpi, Italy) composed by a slightly curved rectangular shape coil fed by a current pulse generator (pulse duration of about 1.3 ms with a current peak of about 1.05 A and 75 Hz repetition frequency), as reported in [13].

**Figure 1:**
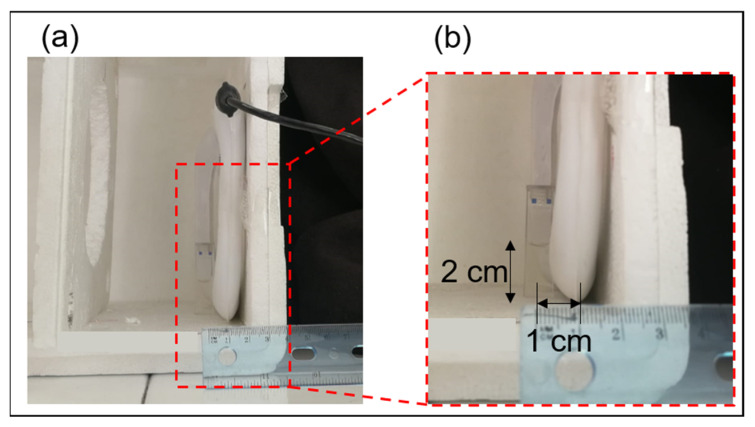
(**a**) Complete experimental setup in place for PEMFs exposures. (**b**) Zoom of the exposure setup to show the distance between coil and cuvette centers.

In order to define the experimental setup in place, numerical simulations of the PEMFs exposure system were performed. A 3D model representing an air box with the magnetic source and the glass cuvette (1.06 × 1.06 × 4.40 cm^3^) filled with the liquid sample (1.0 × 1.0 × 2.0 cm^3^ corresponding to a volume of about 2 mL) was used in Comsol Multiphysics software v 5.5 (COMSOL AB, Stockholm, Sweden). Simulations were carried out solving the magnetic and electric fields module equations at 250 Hz, considering the first lobe of the signal spectral content. Most of the material properties were chosen from the Comsol material library, except for the solution sample relative permittivity of 85 and electrical conductivity of 0.03 s/m, the values of which were confirmed by measurements with a Precision LCR Meter E4980A from Agilent as in [21]. The cuvette was placed at the center of the coil width at a distance of 1 cm as in Figure 1b. The final experimental setup is shown in Figure 1a. Results of the exposure system characterization are reported in terms of magnetic and electric fields in Section 3.1.

The experimental exposure protocol was defined as follows: (1) a diluted (1:25) MLs sample, after SEC purification, is tested in terms of fluorescence intensity to obtain the initial fluorescence reference; (2) the cuvette is filled with the diluted sample and it is placed 1 cm distance between coil-cuvette centers as in Figure 1b; and (3) the PEMFs signal is applied for a total of 3 h, with a sequence of three PEMFs ON (1 h continuously) interlaced with PEMFs OFF (about 10 min) to monitor the fluorescence intensity changes after 1, 2 and 3 h of exposure. The same procedure was used for a not-exposed sample, defined SHAM, but keeping the generator switched off for the entire protocol period. Finally, during each exposure and SHAM procedure, another cuvette is filled with the solution sample and placed on the laboratory desk, far enough from the system to avoid the PEMFs exposure, and this is used as control sample (data not shown). Finally, CLs samples are tested in both the SHAM and exposure procedures. All experiments were performed in triplicate and the results in terms of percentage of dye release (as explained in Section 2.3) were reported as mean ± standard deviation, in Section 3.3.

### 2.5. Temperature Monitoring during the PEMFs Exposure

In order to complete the experimental setup an A325 infrared (IR) thermal camera (Teledyne FLIR LLC, Wilsonville, OR, USA) was used to evaluate possible temperature changes in the experimental bench during PEMFs exposure. The thermal camera is connected to a laptop through an HDMI cable to control the real-time thermal analysis using the FLIR Research IR software, as in [21]. This thermal camera, able to measure temperatures from −20 to 120 °C with an accuracy of ± 2%, is characterized by a thermal sensitivity <0.07 at 30 °C. The temperature acquisition is obtained in terms of images composed by more than 76,000 pixels. Here, the experimental bench temperature was acquired during the 3 h of exposure, setting the frame rate at 16 Hz. The post-elaboration of the recorded data is performed using the FLIR Research IR software and results are reported in Section 3.1. Temperature measurements represent a way to control the entire system and obtain reliable experiments. Moreover, because of the sensitiveness of the MLs sample to any probes, this setup is important in order to avoid any physical interaction with it. Thus, the use of an IR thermal camera allows the acquisition, through non-contact measurements, of thermographic images reporting information not only on the sample but also on the entire experimental bench.

### 2.6. Statistical Analyses

Statistical analyses were performed using two-tailed Student’s t-tests to analyze data. Reported p-values were adjusted using Welsch correction. Significance was reported as (*) for *p* < 0.05, (**) for *p* < 0.01 and (***) for *p* < 0.001.

## 3. Results

### 3.1. Characterization of the Exposure Setup

**Figure 2:**
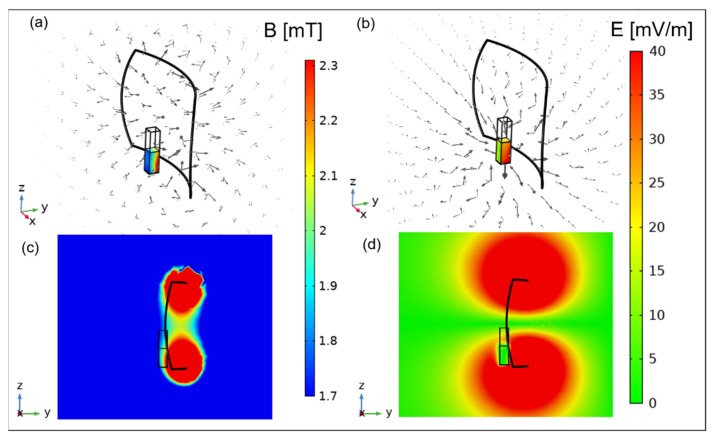
Simulation results of the exposure setup. (**a**) B field spatial distribution in the cuvette and B field arrows; (**b**) E field spatial distribution in the cuvette and E field arrows; (**c**) B field distribution on the central zy plane; (**d**) E field distribution on the central zy cut-plane.

Numerical results on the exposure system characterization are shown in Figure 2. Specifically, the spatial distribution of the generated B field inside the solution sample is reported in Figure 2a, together with the B field arrows with a logarithmic magnitude-controlled length. Here, B field values range from 1.7 to 2.3 mT and the arrows, with a loop shape around the coil, cross the solution sample volume. In panel (b) of Figure 2 the spatial distribution of the induced E field inside the solution sample is shown, together with the E field arrows with a magnitude-controlled distribution. As expected, the induced E field is characterized by low intensity, with values ranging from 0 to 40 mV/m. The E field arrows show the direction of the E field in the simulation box. To have complete information about the magnitude of B and E fields, the spatial distributions of these quantities are reported on the zy plane placed at the center of the simulation box, respectively, in panel (c) and (d) of Figure 2. From Figure 2a,c, it is worth noticing how the generated B field inside the cuvette is not homogeneous, with values from 2.3 mT near the coil and 1.7 mT away from the coil. At the center of the cuvette the B field is about 2 mT. The E field generated in the sample has high values, around 40 mV/m, distributed on the lateral boundaries of the cuvette (Figure 2b), and low values, up to 10 mV/m, generated into the center of the sample as reported in Figure 2b,d. Starting from these simulations, the authors could setup the experimental bench considering an exposure of the solution sample to the low intensity B field of about 2 ± 0.3 mT. This kind of exposure, although using MNPs (embedded in the membrane of liposomes), is considered non-thermal with respect to other non-invasive magnetic stimuli available in the literature, where the goal is the use of superparamagnetic NPs to provide drug delivery nanosystems [22,23,24].

**Figure 3:**
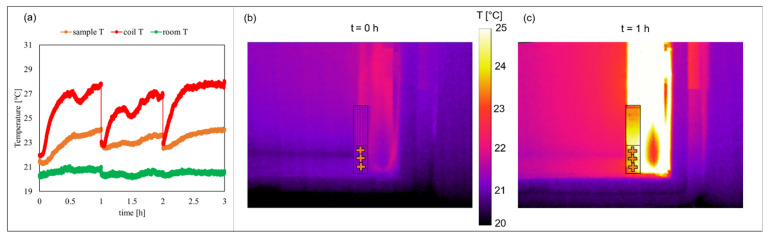
Temperature characterization outcomes. (**a**) Temperature profile in time of the sample (orange curve), the coil (red curve) and the room environment (green curve). Thermographic images of the experimental bench recorded during the PEMFs exposure at t = 0 h (**b**) and t = 1 h (**c**). Selected cursors on the sample are reported as orange crosses.

In this regard, thermal information has been analyzed considering the temperature distribution in time for: (1) the MLs sample; (2) the commercial coil; and (3) the room environment. For each of them, three different cursors have been selected and their statistical temperature data have been recorded. Panel (a) of Figure 3 shows the temperature profile in time recorded in the sample (orange curve), on the coil (red curve) and in the room environment (green curve), reported as the mean value over the three selected cursors. The sample temperature slightly changes in time ΔT= 1.25 ± 0.75 °C during each hour of exposure confirming a non-thermal PEMFs exposure. An example of thermal images is reported in panels (b) and (c), where images are shown at the beginning of the experiment (PEMFs ON t = 0 h) and after 1 h. The edges of cuvette and sample inside it are underlined with black lines and the three cursors selected for the analysis of the sample temperature are reported as orange crosses. From these images it is evident how the coil temperature changes in time, determining an increase of temperature around it. Due to the flowing current, the coil temperature increases up to 5.1 ± 0.8 °C during each hour of the exposure experiment, while the room temperature fluctuates by about 0.5 °C around 20 °C (see Figure 3a red and green curves respectively). Thanks to the heat exchange with the room environment, the sample temperature presents only a mild increase in time, as already discussed.

### 3.2. Physicochemical Characterization of Vesicles

The optimal lipids/MNPs molar ratio was chosen as reported by Bothun [25]. Generally, the more MNPs added to the lipids the more MNPs aggregate with each other, rather than assemble in the hybrid vesicles. The lipids/MNPs ratio was considered as optimal when higher amounts of MNPs resulted in the formation of aggregates with a diameter higher than the membrane thickness. When the TLE was considered, a visible high loss of MNPs was observed during the extrusion process and undesired aggregate formation took place, regardless of the lipid/MNP molar ratio (See Supplementary Figure S1). The loss of magnetic material makes the TLE method followed by extrusion not suitable for MLs preparation with our oleic acid-coated MNPs. For this reason, we continued with REV method, which leads to the direct formation of unilamellar vesicles without the need of the extrusion step. Results obtained by DLS measurements and using the Zetasizer Pro, are reported in Table 1, where size, PDI and Zeta Potential of the three kinds of MLs as well as CLs are reported and, depending on the phospholipid composition chosen, named as Gel, Liquid Disorder and Liquid Order.

The data highlight that such an approach is always suitable to generate homogenous MLs population with a size < 100 nm and PdI < 0.2 using hydrophobic MNPs with a superficial layer of oleic acid, similarly to what happens for CLs.

The slight increase in the average MLs size with respect to CLs is consistent with the morphologic deformation of the bilayer that, in the immediate vicinity of the MNPs, could wrap around them. The lipid arrangement during the REV technique was not affected even when cholesterol (chol) was added in order to obtain the so-called Liquid Order MLs, that is, a vesicle which is characterized by an intermediate rigidity of the bilayer with respect to the Gel MLs and the Liquid Disorder MLs.

**Figure 4:**
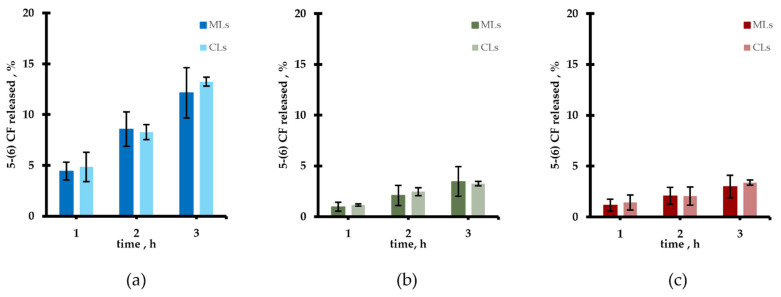
Percentage of spontaneous release from MLs and CLs of: (**a**) Liquid Disorder Liposomes, (**b**) Gel Liposomes, (**c**) Liquid Order Liposomes.

Regarding the release properties of the different MLs samples, the spontaneous leakage of the fluorescent probe 5-(6) CF was monitored vs time and compared with results obtained with CLs and reported in Figure 4; as evident no spontaneous release difference between all the MLs samples and their corresponding CLs occurred up to three hours. While for the Liquid Disorder Liposomes (Figure 4a), the spontaneous release is attested around 13% at 3 h of monitoring, for the remaining two (Figure 4b,c), such release is limited to no more than 4%. As expected, cholesterol added for the Liquid Order Liposomes, induces conformational order of the fluid state lipid chains, and reduces the entity of the spontaneous leakage, making it similar to that of the Gel ones. Similar release from MLs and CLs is consistent with the fact that oleic acid-coated MNPs did not affect the bilayer permeation of both high-Tm and low-Tm phosphatidylcholines vesicles even when cholesterol was added to these last ones. All samples were stable upon storage at 4 °C for at least 24 h.

### 3.3. Release in Exposure Conditions

The release of 5-(6) CF under PEMFs exposure was monitored at 25 °C for 3 h to study the MNPs effect on the three different bilayer types. During the PEMFs exposure, MLs samples did not show any significant increase in temperature in the bulk solution; it was always ΔT < 1.25 °C, due to the low level of PEMFs exposure (Section 3.1). At the same time, Liquid Disorder, Liquid Order and Gel MLs were tested in SHAM. Furthermore, to exclude any possible direct PEMFs effects on the bilayer itself, dye release was monitored under the same conditions for the CLs.

**Figure 5:**
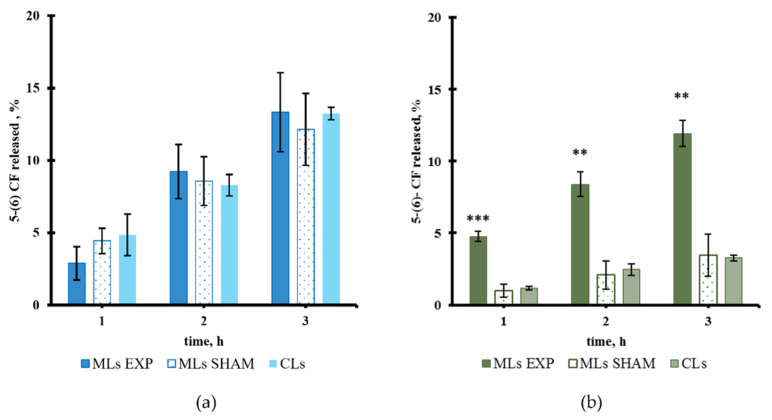
Percentage of release from MLs and CLs during PEMFs exposure and SHAM condition: (**a**) Liquid Disorder MLs (**b**) Gel MLs. (**) *p* < 0.01 and (***) *p* < 0.001.

As shown in Figure 5a, no field-induced release of the 5-(6) CF was observed among the different Liquid Disorder vesicles (MLs, SHAM and CLs) at any of the observation times. From Figure 5b, it is worth noticing that the application of PEMFs induced a significant increase in the dye release rate (around 12% at 3 h) from Gel MLs with respect to the almost 3% of the SHAM and the CLs. These results demonstrate that at 25 °C, below the gel (Lβ) to liquid-crystalline (Lα) phase transition temperature (Tm = 52.6 °C) of the rigid HSPC, MNPs were able to move and the permeability of the rigid bilayer was affected by their motion, due to the PEMFs application.

**Figure 6:**
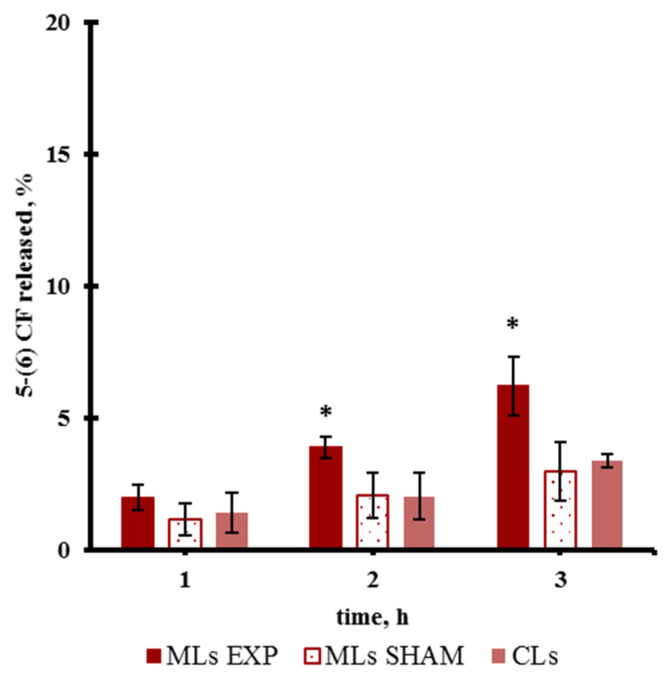
Release from Liquid Order MLs and CLs during PEMFs exposure and SHAM condition. (*) *p* < 0.05.

Finally, Figure 6 reports a slightly increased release after PEMFs exposure for Liquid Order MLs with respect to SHAM and CLs.

**Figure 7:**
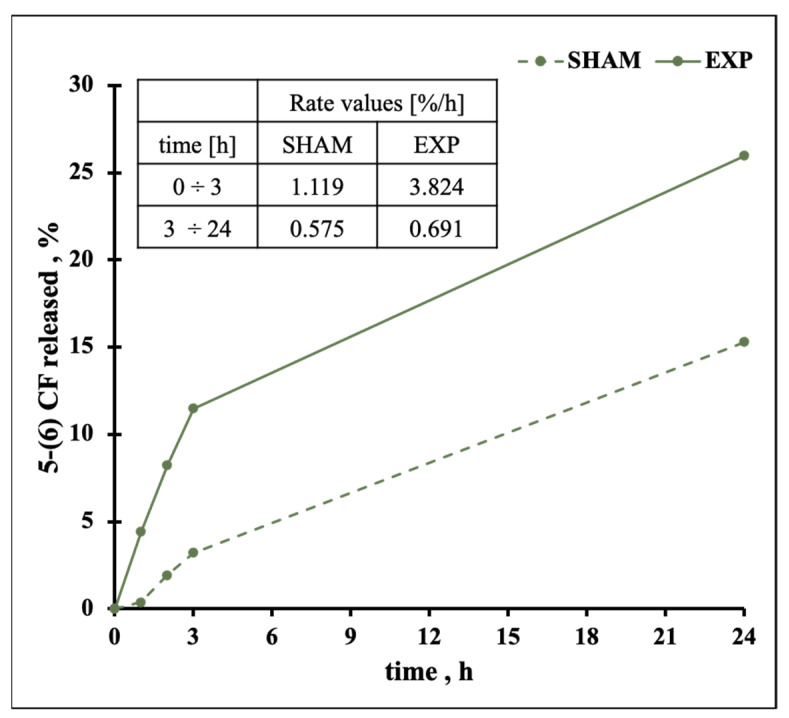
Release from Gel MLs during PEMFs exposure and SHAM condition (3 h) and after 24 h. The inset shows the rate of release for the first 3 h of exposure and for the remaining 24 h without PEMFs.

In order to further investigate the effect of PEMFs on gel liposomes, the release was monitored for 24 h following the 3 h of exposure as reported in Figure 7. Once the field was switched off, the rate of release decreased, becoming comparable to the one of the SHAMs and indicating that Gel MLs can recover their physical properties (see the Table inset comparing the rate values).

**Figure 8:**
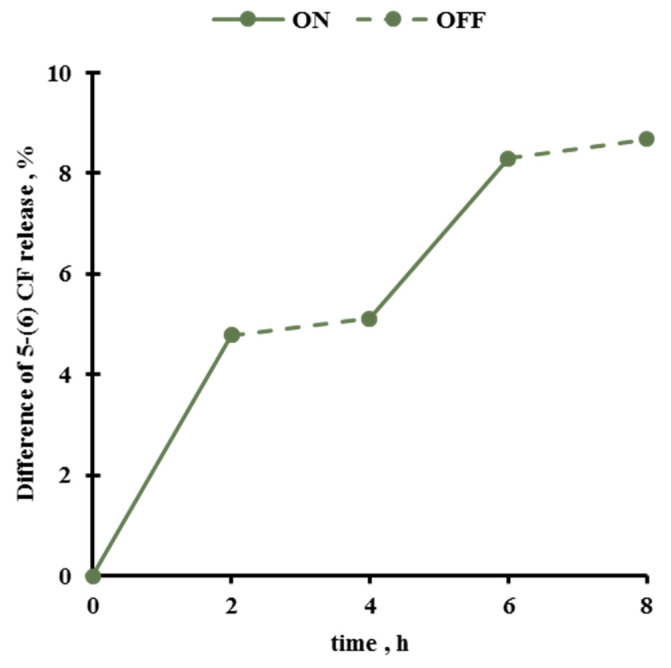
Release from Gel MLs during PEMFs exposure in ON-OFF modality.

These findings suggest that the content released from the Gel MLs could be triggered repetitively by switching ON and OFF the PEMFs stimulus. Figure 8 reports the response of Gel MLs triggered by switching the PEMFs ON (2 h) and OFF (2 h). In particular, the difference between the exposed and SHAM release is reported over time with a value of almost 5% due to the first turn and 3% during the second one; notably no release can be appreciated during both the OFF periods.

## 4. Discussion

When an external stimulus is used as a trigger to control drug release from liposomes, vesicles should be formulated evaluating the phospholipids peculiarities to obtain sensitive drug delivery systems. The responsiveness of selected bilayers will allow the release of therapeutics to be triggered on-demand.

In this regard, the liposome bilayers’ liquid or gel phase, according to their composition and temperature, can be of importance, and Tm temperature can play a role. At temperatures above Tm, the lipid bilayer exists in the liquid phase, characterized by a relatively high degree of lateral mobility of individual lipids within the bilayer. On the contrary, at temperatures below Tm, the lipid bilayer exists in the gel phase characterized by closely packed lipids. Tm influences directly the order and the mechanical stability of the lipid bilayers and depends on many factors e.g., acyl chain length, saturation state, charge, and head-group species. Since the lateral organization of the lipid molecules determines the mechanical properties of the bilayers, such properties need to be carefully considered in the proper design of on-demand drug delivery systems based on liposomes.

Among all investigated stimuli-responsive liposomes, early efforts focused on the use of embedded magnetic nanoparticles (MNPs) and an external magnetic field as a trigger to control drug delivery [4,11,12,13,14,15,16,17,18,19]. Usually, magnetic hyperthermia is used to trigger release of drugs from MLs. This approach exploits MNPs as nano-heaters to produce a modification in the physical state and consequently to the permeability of MLs. For this reason, the successful application of the magneto-thermal approach strictly requires the use of temperature-sensitive liposomes, with a Tm in the range 39 °C to 42 °C. The physical mechanism behind this approach involves heat dissipation by the MNPs exposed to the magnetic field that produces an increase in the permeability of MLs membranes or a real phase transition. To this end, a proper selection of the lipid (or the lipid mixture) is essential to obtain a bilayer with the appropriate Tm in order to gain this final effect.

More recently, it has been observed that it is possible to obtain a controlled drug release from MLs even in non-heating conditions, using low AMF or PEMFs of appropriate intensity and frequency [12,13,14]. Under these conditions, MNPs act as magneto-mechanical actuators. In this case, the physical mechanism does not involve heat dissipation by the MNPs, but their vibration or rotation produces a mechanical destabilization of lipid membranes of MLs. The efficacy of this approach has been proved with MLs containing hydrophilic MNPs.

When liposomes embed hydrophobic MNPs within their bilayer it becomes even more crucial to understand whether the nanoparticles can still move in the lipid microenvironment under the effect of non-thermal PEMFs. In this context it is of importance to understand the influence of phospholipid composition, lateral packing and organization on the effectiveness of magneto-mechanical actuation. Indeed, depending on the type of lipids used to produce MLs, the responsiveness of the carrier could be enhanced or reduced. Since non-lipid components of phospholipid vesicles may be able to modulate the physicochemical properties of lipid membranes, such as their main transition temperature, thermograms of the three different bilayers are recorded according to [26] and reported in Supplementary Material as Figure S3. Results showed only slight variations in the thermal behavior of the samples, which do not substantially modify the thermotropic characteristics of the gel, liquid order and liquid disorder hybrid membrane with respect to the corresponding conventional membrane.

**Figure 9:**
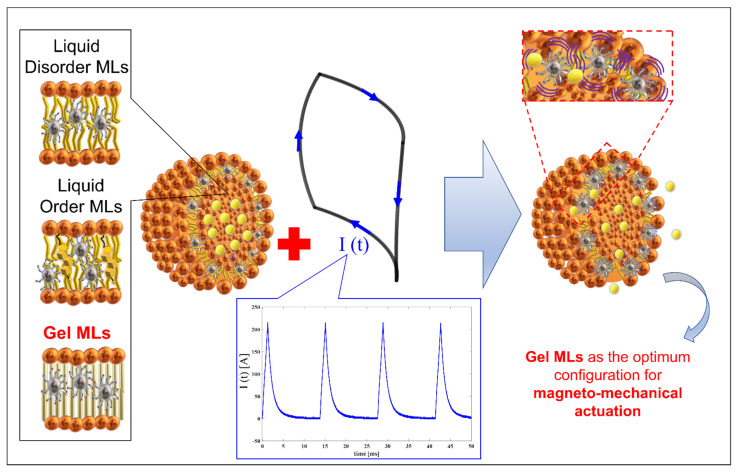
Scheme of the hypothesized mechanism of magneto-mechanical actuation based on PEMFs coupling with MLs. The current flowing in the coil produces the B field, which promotes oscillation of MNPs inside the bilayer of Gel MLs (organized with a rigid order packing) and finally destabilization of the membrane vesicle with release of its content.

The hypothesis tested in this paper, and schematically reproduced in Figure 9, is that oscillating MNPs embedded in a gel state bilayer could affect the rigid ordered packing of the lipids and could trigger the cargo release, whereas in a liquid-crystalline membrane, defects created by mechanical motion of the MNPs could be recovered, thus resulting in a decreased release of cargo.

MNPs encapsulation occurs when lipid vesicles self-assemble from the collapsed gel-like state formed during the REV procedure. Considering the hydrophobic nature of the MNPs coating and the drug release ability of the obtained liposomes/MNPs system, the MNPs are somehow incorporated within the bilayer of the final construct.

Under the action of PEMFs, MNPs seem to be able to move in the ordered rigid bilayer of the Gel MLs and their movements perturb the lipid packing order, increasing the bilayer permeability, as assessed by the higher release of the co-entrapped fluorescent dye (Figure 5b). On the other hand, no PEMFs-induced release of the dye was observed from the Liquid Disorder MLs (Figure 5a). These results suggest that lipid packing is very important to magneto mechanical responsiveness. We speculate that defects in the packing of the liquid disordered bilayer could be neglected because of the elastic deformation of the fluid state membrane, nullifying the mechanical stress. Conversely, the entrapped hydrophobic MNPs can effectively improve the bilayer permeability of Gel MLs as a consequence of their motions under PEMFs exposure, without affecting the geometrical properties of the liposomes as revealed by DLS (see Supplementary Materials Figure S2). Considering these results, the release from the Gel MLs can be explained by the mechanical motions of hydrophobic MNPs and modification of the phospholipids arrangements rather than the destruction of the liposome structure.

The Liquid Disorder MLs that did not respond to PEMFs application were made more rigid by adding cholesterol (named here Liquid Order MLs). After the modification of the lipid packing to a more ordered state, the response of the MLs was modified, obtaining a slight release after PEMFs exposure. As a whole our data suggest that the release triggered by the PEMFs magneto mechanical approach depend strongly on the rigidity of the bilayer.

## 5. Conclusions

In this work, thanks to the REV technique, we produced MLs with different lipid compositions in order to obtain Gel, Liquid Order and Liquid Disorder vesicles embedding hydrophobic MNPs within their membrane. To probe their membrane permeation and their release behavior, a water-soluble fluorescent dye was loaded in MLs. The exposure to PEMFs was carried out for a total of 3 h as an exposure protocol compatible with medical low-level magnetic field treatments. The release mechanism seems to be related to the mechanical stress on the liposome membrane caused by MNPs movements in its proximity. This study allowed the evaluation of the role of the phospholipid packing on the capacity of MLs to respond to the external application of PEMFs stimulus by means of MNPs mechanical motion. The responsiveness of the three types of MLs critically depends on the stiffness of the phospholipid membrane. Indeed, only Gel and Liquid Order MLs exhibited a modification of the bilayer permeability when exposed to PEMFs. On the contrary, MLs characterized by a Liquid Disorder bilayer are able to dampen the perturbation produced by the MNPs movements, due to their fluidity. These results are the basis for a proper selection of lipid composition of MLs bilayers that will encapsulate hydrophobic MNPs in it and will be able to efficiently respond “on-demand” when exposed to low-intensity magnetic fields.

## Figures and Tables

**Table 1:** Characterization results based on ^a^ intensity size distribution model, ^b^ polydispersity index, ^c^ zeta potential in HEPES buffer at pH = 7.4.

Sample	Hydrodynamic Diameter ^a^ Mean ± SD (nm)	PdI ^b^ Mean ± SD	Zeta Potential ^c^ ± SD
Gel MLs	99.4 ± 0.2	0.106 ± 0.083	−9.58 ± 0.85 mV
Liquid Disorder MLs	84.7 ± 0.3	0.105 ± 0.071	−9.60 ± 1.95 mV
Liquid Order MLs	93.2 ± 0.7	0.199 ± 0.061	−15.20 ± 0.60 mV
Gel CLs	76.0 ± 1.9	0.106 ± 0.036	−8.31 ± 0.65 mV
Liquid Disorder CLs	80.9 ± 0.6	0.226 ± 0.156	−7.69 ± 0.75 mV
Liquid Order CLs	79.2 ± 0.1	± 0.042	−14.9 ± 1.11 mV

## Data Availability

The data presented in this study are available upon request to the corresponding author.

## Supplementary Materials

The following are available online at https://www.mdpi.com/article/10.3390/pharmaceutics13101712/s1, Figure S1: Visible MNPs aggregate formation on membrane filters with consequent loss of magnetic material, Figure S2: DLS analysis of MLs before (a) and after (b) PEMFs exposure, Figure S3: DSC analysis of Liquid Disorder (a), Liquid Order (b) and Gel (c), in the presence or not of MNPs.
