# Supplementary material for: The Impact of Bilayer Rigidity on the Release from Magnetoliposomes Vesicles Controlled by PEMFs
Source: Pharmaceutics. 2021 Oct 16;13(10):1712. doi: 10.3390/pharmaceutics13101712 (PMC8538647; doi:10.3390/pharmaceutics13101712)

pharmaceutics

MDPI

The impact of bilayer rigidity on the release from magnetolipsomes vesicles controlled by PEMFs

Jordan Trilli [1,*], Laura Caramazza [2,3,*], Patrizia Paolicelli [1], Maria Antonietta Casadei [1], Micaela Liberti [2,3], Francesca Apollonio [2,3,], Stefania Petralito [1]**

[1] Department of Drug Chemistry and Technologies, Sapienza University of Rome, 00185, Rome, Italy; jordan.trilli@uniroma1.it; patrizia.paolicelli@uniroma1.it; mariaantonietta.casadei@uniroma1.it; stefania.petralito@uniroma1.it
[2] ICEmB at DIET, Sapienza University of Rome, 00184, Rome, Italy; laura.caramazza@uniroma1.it; micaela.liberti@uniroma1.it; francesca.apollonio@uniroma1.it
[3] Center for Life Nano- & Neuro-Science, Fondazione Istituto Italiano di Tecnologia (IIT), 00161, Rome, Italy; laura.caramazza@iit.it
* These authors contributed equally to this work
** Correspondence: francesca.apollonio@uniroma1.it;

Thin Layer Evaporation Techniques (TLE)

For TLE technique, liposomes were prepared as previously reported by Petralito et al., with slight modification [16]. Briefly, 40.0 mg of Egg-PC were dissolved in 3 mL of chloroform (supplemented with 0.5 mg of MNPs for MLs) and evaporated to form a thin film. The thin film was hydrated with 5 mL of HEPES buffer solution (10 mM, pH 7.4), agitated with a vortex and then extruded through membrane filters having 0.8, 0.4 and 0.2 μm pores size.

Figure S1. Visible MNPs aggregate formation on membrane filters with consequent loss of magnetic material.

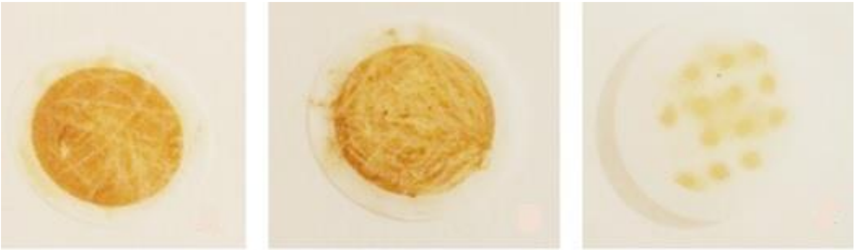

*Figure S2. DLS analysis of MLs before (**a**) and after (**b**) PEMFs exposure.*

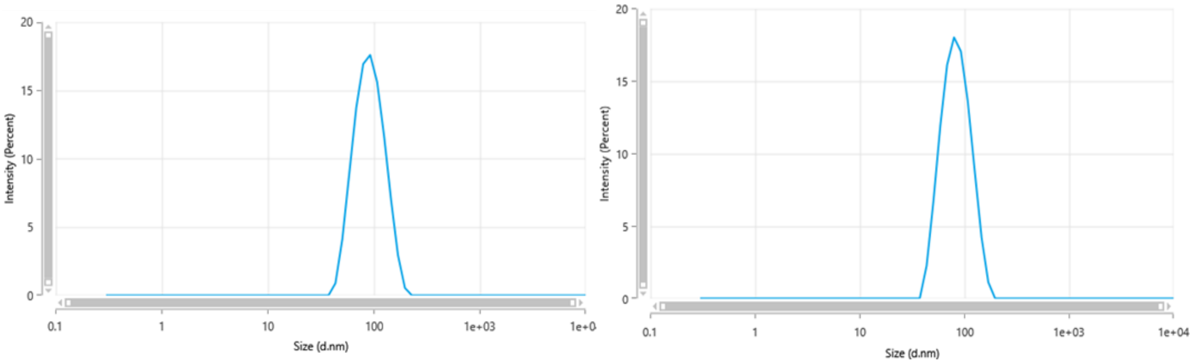

(a) (b)

Differential scanning calorimetry (DSC) charactrization

Samples (5 mg) of the different mixtures were weighed in sealable aluminum pans, 20 µl of distilled water was added, and then the pans were hermetically sealed. At least three heating/cooling cycles in a temperature range from 30 to 60 °C or from -20 to 25 °C under nitrogen flow (20 mL/min) were performed with a scan rate of 2 °C/min, on each sample. An empty aluminum pan was used as reference. This method was used to evaluate all membrane phase transitions and how the presence of MNPs in the bilayer influences this parameter.

Figure S3. DSC analysis of Liquid Disorder (a), Liquid Order (b) and Gel (c), in the presence or not of MNPs.

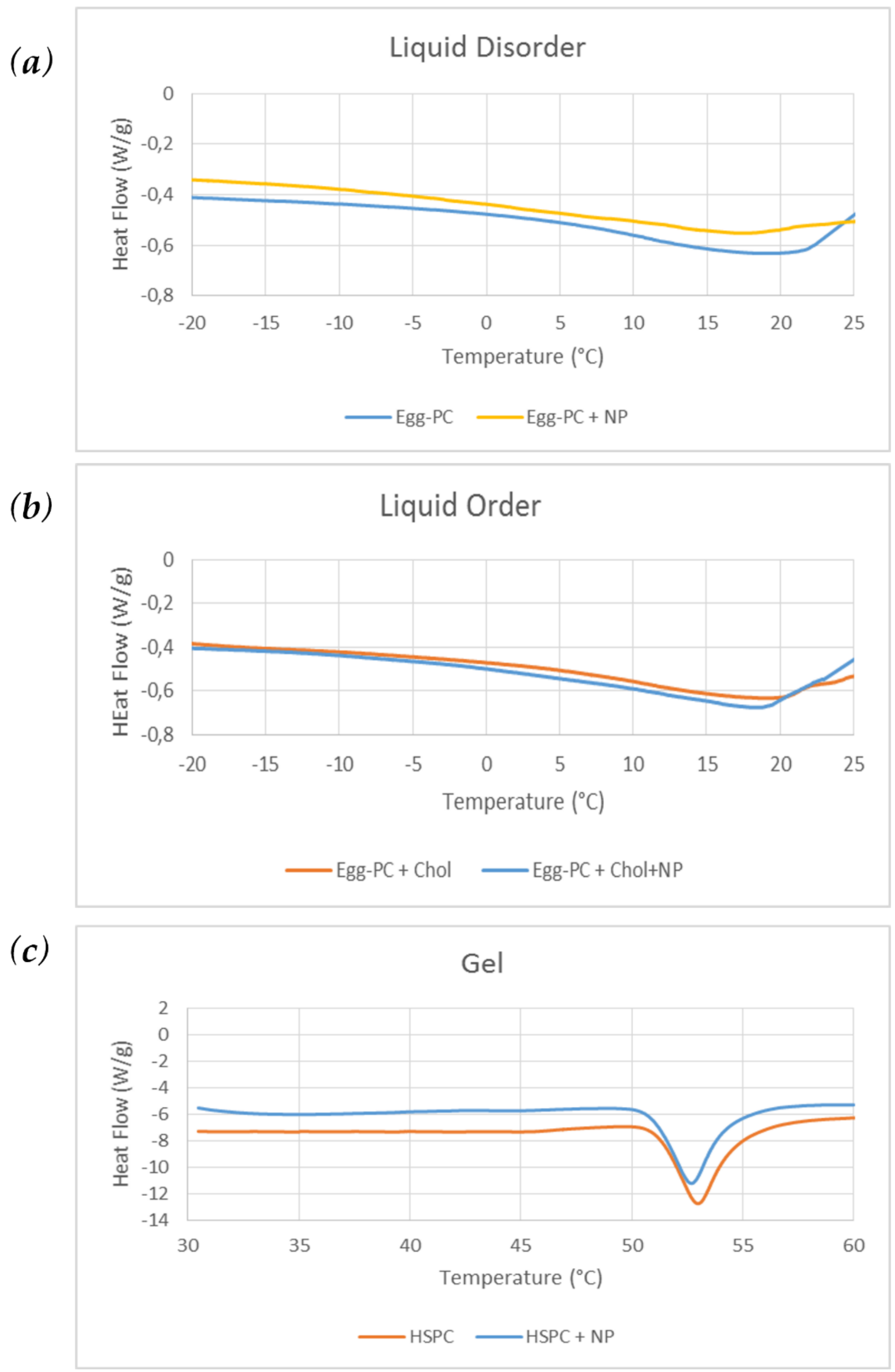

Supplement: Supplementary file 1 [file pharmaceutics-13-01712-s001.zip › pharmaceutics-1330192-supplementary.pdf]
